# Quantifying the Attrition of Health System Contact into Quality-Adjusted Screening Coverage for Acute Malnutrition among Children in Ethiopia

**DOI:** 10.1016/j.cdnut.2026.107711

**Published:** 2026-05-06

**Authors:** Tarik Taye, Seifu Hagos, Samson Gebremedhin, Alinoor Mohamed, Mark Spigt, Beshada Rago, Beza Yilma

**Affiliations:** 1 Department of Family Medicine, School CAPHRI, Care and Public Health Research Institute, Maastricht University, Maastricht, The Netherlands; 2 Department of Nutrition and Dietetics, School of Public Health, College of Health Sciences, Addis Ababa University, Addis Ababa, Ethiopia; 3 Department of Community Medicine, General Practice Research Unit, University of Tromsø, Tromsø, Norway

**Keywords:** acute malnutrition, Ethiopia, Health Extension Program, quality, screening, children under 5

## Abstract

**Background:**

High-quality screening for malnutrition is a critical entry point for children with acute malnutrition to access lifesaving nutritional care, treatment, and support. However, the contacts with Community Health Workers are suboptimal, and evidence of the quality of screening for acute malnutrition at the community level is limited.

**Objectives:**

This study examines screening practices for acute malnutrition among children 6 to 59 mo old to provide an in-depth insight on the quality of screening in Ethiopia.

**Methods:**

We used data from a community-based cross-sectional study to investigate the quality-adjusted screening coverage of acute malnutrition among children 6 to 59 mo old in 6 selected regions of Ethiopia. A multistage cluster sampling method was employed to select the study participants. A quality index was constructed using need-based variables to adjust for the quality of screening. Caregivers of the children were interviewed to ascertain child nutritional screening practices during the 3 mo preceding data collection and estimate contact coverage, crude screening coverage, and quality-adjusted screening coverage.

**Results:**

A total of 1905 children, 6 to 59 mo old, were enrolled in the study. Less than half (39.3%) of the children in our study were visited by a Community Health Worker in the preceding 3 mo, of which, 72% were checked for nutritional status. When the nutritional screening was adjusted for quality, only 40.4% of the children contacted had the health benefits of the nutritional screening.

**Conclusions:**

The study reveals a critical gap in the contact between Community Health Workers and children aged 6 to 59 mo. With only 4 of 10 children visited by Community Health Workers receiving optimal nutritional screening, many children miss the health benefits from routine nutritional screening because of inadequate screening or none at all. To close the gap, stakeholders should prioritize monitoring the quality and performance of screening for acute malnutrition in Ethiopia.

## Introduction

Early detection of acute malnutrition is crucial, according to the WHO, as it enables prompt treatment and prevents severe consequences [1]. WHO recommends member states to roll out a community-based nutrition screening program using Community Health Workers (CHWs) and trained community members through routinely measuring mid-upper arm circumference (MUAC) and checking children for bilateral pitting edema. Globally, 45% of children <5 y are affected by wasting, a MUAC <12.5 cm or a weight for height <−2 z-score in children 6 to 59 mo [2]. More than a quarter of the global wasting burden among children under 5 y is in Africa [3]. Following on WHO’s recommendations, several low- and middle-income countries (LMICs) shifted from traditional facility-based management of acute malnutrition to community-based management of acute malnutrition (CMAM), resulting in wider coverage, higher recovery, and lower mortality rate [4]. Ethiopia is one of the countries that adopted WHO’s recommendations for the management of acute malnutrition [5].

Household/community visits by CHWs are associated with higher utilization of essential maternal and child health services. In Ethiopia, mothers with frequent visits from Health Extension Workers (HEWs) are 30% more likely to seek care [6]. The national nutritional screening program can prevent and reverse acute malnutrition outcomes through early identification, support, and timely management of comorbidities. CMAM in Ethiopia prevented nearly half a million child deaths between 2008 and 2020 [7,8]. However, CHW contacts and visits are suboptimal, and evidence on the quality of community-level screening for acute malnutrition is limited [9].

In addition, contact with CHWs is essential but insufficient to ensure children experience the benefits of primary healthcare services [10]. Countries are also encouraged to monitor if lifesaving interventions such as screening for acute malnutrition are reaching all children at scale with an acceptable quality, breaking economic and geographic barriers [11]. This is measured by assessing the coverage of the acute malnutrition programs using proportions of eligible children who were reached by the national nutrition program. The Ministry of Health (MoH), Ethiopia, in its Health Sector Medium-term Development and Investment Plan 2023/2024 to 2025/2026, sets out a target to improve coverage of growth monitoring from the baseline 63% to 75% by the end of 2026 [12].

Many countries in Sub-Saharan Africa (SSA), including Ethiopia, fall short of reaching their targets for coverage because of barriers ranging from poor awareness by the community to poor health system preparedness [13]. A study in Ghana showed that only 6% and 29% children were reached by CMAM in urban and rural facilities, respectively [14]. Similar studies in Burkina Faso, Chad, Democratic Republic of Congo, Niger, and Somalia reported an alarmingly low proportion of children, <20%, benefiting from CMAM across all the countries [15]. Another study covering 21 countries from SSA also resulted in a 38.3% mean coverage of CMAM [13]. In addition to similarities in trends of low program coverage, the studies reported coverage results focusing on the treatment of acute malnutrition, with little emphasis on screening coverage, which is a critical entry point for treatment. This was also reflected in the National Guideline for the Management of Acute Malnutrition in Ethiopia, which provides 4 key program indicators to periodically monitor performance; however, all 4 indicators are related to treatment outcomes of children who are enrolled for severe acute malnutrition or moderate acute malnutrition treatment [2].

Hence, this study examines screening practices to provide in-depth insight on the quality of screening for acute malnutrition, which is a critical entry point to link children with lifesaving nutritional care, treatment, and support services in Ethiopia. Assessing the quality-adjusted screening coverage (QASC) will fill the gap in understanding the barriers to an effective nutrition screening program for children <5 y.

## Methods

### Study design

A community-based cross-sectional study design was employed to investigate the QASC of acute malnutrition in 6 selected regions of Ethiopia. The experience of nutritional screening among children 6 to 59 mo old living in the study districts was assessed using a survey by enrolling their care takers as the study participants. Data were collected in June 2023 as part of the baseline assessment for implementation research on Ethiopia’s national Integrated Management of Acute Malnutrition (IMAM) program. The Federal Democratic Republic of Ethiopia is administratively divided into 12 regional states and 2 city administrations, with 77% of the population living in rural areas [12]. This study was conducted in 18 districts selected from 6 regions of Ethiopia, namely Afar, Amhara, Oromia, Somali, and South Ethiopia regions (Table 1).

**TABLE 1:** Regions and districts covered in the study

	Regions					
	Afar	Amhara	Oromia	Somali	Sidama	SNNP
Districts	Mile	Dalanta	Girawa	Adadke	Aleta Chuko	Zala
	Chifra	Meket	Bedeno	Mustahil	Shebedino	Uba Dhebresahay
	Adaar	Dawunt	Deder	Gode	Dale	Kucha

Abbreviation: SNNP, Southern Nations Nationalities and Peoples'.

### Sample size and sampling methods

Sample size for the participants was calculated using OpenEpi, version 3, an open-source online calculator. The assumptions considered were a confidence limit of ±5%, design effect of 2 [16], an anticipated 63% crude screening coverage [12], and a QASC of 50%. Multiple sample size calculations were carried out for the 4 research objectives, and the highest sample size estimated was 768. However, for practical purposes, all the 1905 children from the dataset are included in the analysis of this study.

A multistage cluster sampling method was employed to select the study participants. Selection of regions and districts for the study followed a convenient sampling technique, factoring in the availability of a functional CMAM or IMAM program in the district. Selection of study participants involved the selection of 3 Kebele’s (the lowest administrative unit in Ethiopia) for each of the 18 districts using stratified random sampling. After the selection of Kebeles, existing 1 to 30 household networks were listed, 5 networks were randomly selected for each Kebele; finally, 8 households with children 6 to 59 mo were selected from each of the 1 to 30 household networks using a random walk method.

### Data collection and analysis

Data collection was managed by trained and experienced enumerators and supervisors. To ensure data quality, all the data collectors and supervisors went through a 4-d training before deployment. Data were collected using a structured and pretested survey questionnaire developed based on the standards from the national guideline for the management of acute malnutrition [2]. The questionnaire was first developed in English and translated to the respective local languages of the study localities (Amharic, Afan Oromo, and Somali). Survey data were collected using an Open Data Kit to ensure real-time quality data collection, cleaning, and monitoring.

### Data management and analysis

**FIGURE 1:**
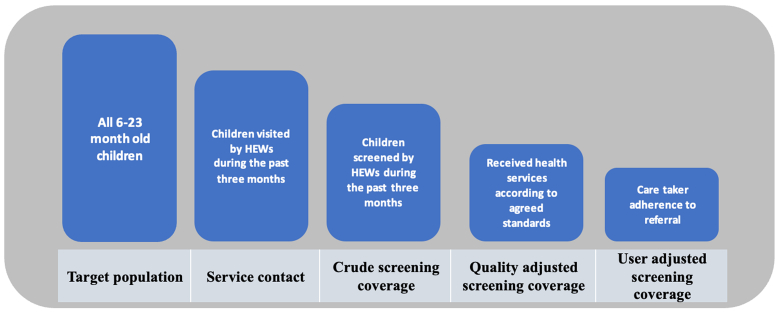
Loss of children along the cascade of screening and case identification.

The data were exported and analyzed for frequency distribution and appropriate measures of central tendency using STATA V18.0 (StataCorp LLC). A coverage cascade framework was adopted to develop a cascade for screening of children for acute malnutrition [17]. Figure 1 illustrates the potential loss of health benefits using an adopted and modified cascade for screening of acute malnutrition that can be used to track QASC of acute malnutrition.

### Index creation and analysis

Strategies outlined for screening by the Ethiopian national guideline for the management of acute malnutrition was used to construct the quality index. According to the guideline, case finding involves house-to-house screening visits by HEWs, community screening during integrated outreach days, health facility visits, and through self-referrals [2]. Moreover, the guideline instructs the following essential actions to be carried out at the community level during screening for children between 6 and 59 mo of age:

(i) MUAC measurement
(ii) Assessment of bilateral pitting edema
(iii) Referral for nutritional supplements and associated medical conditions
(iv) Referral for food aid
(v) Follow-up

To assess contact, care takers of children enrolled in the study were asked if they were visited by an HEW in the past 3 mo. The visit did not have to be specific to nutritional screening, given that HEWs provide additional health promotion and disease prevention services targeting women and children. Frequency distribution was used to estimate the contact and crude screening coverage.

Equation 1: contact coverage

$$\text{Contact}\;\text{coverage}=\frac{\text{Number}\;\text{of}\;\text{children}\;6-59\;\text{mo}\;\text{who}\;\text{had}\;1\;\text{or}\;\text{more}\;\text{visits}\;\text{from}\;\text{an}\;\text{HEW}\;\text{in}\;\text{the}\;\text{past}\;3\;\text{mo}}{\text{Total}\;\text{number}\;\text{of}\;\text{children}\;6-59\;\text{mo}}$$

To estimate the crude screening coverage, care takers were asked if anyone (excluding the care taker) checked the nutritional status of their child by taking MUAC or weight or height measurement, or pressing both feet/lower limbs with thumbs to check swelling? This will qualify if there was any form of nutritional screening happening in the past 3 mo.

Equation 2: crude screening coverage

$$\text{Crude}\;\text{screening}\;\text{coverage}=\frac{\text{Number}\;\text{of}\;\text{children}\;\text{who}\;\text{received}\geq 1\;\text{nutritional}\;\text{screening}\;\text{in}\;\text{the}\;\text{past}\;3\;\text{mo}}{\text{Children}\;6-59\;\text{mo}\;\text{who}\;\text{had}\geq 1\;\text{visits}\;\text{from}\;\text{an}\;\text{HEW}\;\text{in}\;\text{the}\;\text{past}\;3\;\text{mon}}$$

As per the recommendations of the national guideline for the Management of Acute Malnutrition in Ethiopia [2], a Quality Index was developed using need-based quality variables for screening.

Depending on the child’s nutritional status, caretakers were asked specific questions on child nutritional screening methods (MUAC, edema checks), communication of screening results, nutrition education, and linkage to care. On the basis of the responses for the above questions and based on the nutritional status of the child, the following need-based variables were developed:

(vi) MUAC measurement
(vii) Checking for bilateral pitting edema
(viii) Informing nutritional status
(ix) Advise to maintain feeding for children with good nutritional status
(x) Advise to improve feeding for children with poor nutritional status
(xi) Advised (other)
(xii) Referral of children with poor nutritional status for nutritional and healthcare and
(xiii) Referral of care takers of children with poor nutritional status to food aid services based on the national guidelines.

Individual frequencies were calculated for each of the selected need-based variables based on the applicability of the respective variables. Finally, the quality index was generated by computing the weighted mean of the individual frequencies of the need-based variables, and the QASC was estimated by using the following formula adopted from Shengelia et al. [18].

Equation 3: QASC

$$\text{QASC}=\text{Quality}\;\text{index}\times \frac{\text{Number}\;\text{of}\;\text{children}\;6-59\;\text{mo}\;\text{screened}}{\text{Children}\;6-59\;\text{who}\;\text{had}\geq 1\;\text{visits}\;\text{from}\;\text{an}\;\text{HEW}\;\text{in}\;\text{the}\;\text{past}\;3\;\text{mo}}$$

### Ethical considerations

Ethical approval was secured from the Institutional Review Board of Addis Ababa University under protocol number 116/22/SPH. The ethical considerations involved in this study were carefully observed to protect the rights, welfare, and privacy of the participants and in compliance with national and international ethical standards at all stages of the study. Furthermore, administrative permission was also granted from the Federal MoH, respective Regional Health Bureaus, and health facilities before data collection. In addition, informed consent was taken from all parents and caretakers before enrolment in the study.

## Results

A total of 1905 children 6 to 59 mo of age were included in the study. Both boys and girls were equally represented, with the majority (67.4%) being over 2 y old. The majority of the caretakers interviewed were mothers of the children (95.2%), and half of the caretakers were within the age group of 25 to 34 y. In relation to their educational status, half of the caretakers had no formal education, and a large proportion (93.6%) of them were married (Table 2).

**TABLE 2:** Sociodemographic characteristics of the participants

Variables	Number (*n* = 1975)	Percent (95% CI)
Child		
Age (mo)		
6–11	136	7.1(6.2, 8.1
12–23	483	25.3(23.1, 27.7
24–59	1286	67.4(64.6, 70.1
Sex		
Boy	926	48.6
Girl	979	51.4
Caretaker		
Relationship to the child		
Mother	1813	95.2
Father	25	1.3
Other primary care taker	67	3.5
Total	1905	100
Sex		
Male	105	5.5
Female	1800	94.5
Age (y)		
15–24	463	24.3 (22.2, 26.5
25–34	936	49.1 (46.5, 51.7
35–44	456	23.9 (21.3, 26.7
45 and above	50	2.6 (1.8, 3.7
Total	1905	100
Highest educational status achieved		
No formal education	968	50.8
Primary education	751	39.4
Secondary education	171	9.0
Tertiary education	15	0.8
Marital status		
Single	10	0.5
Married	1783	93.6
Separated	32	1.7
Divorced	30	1.6
Widowed	50	2.6

Abbreviation: CI, confidence interval.

With the exception of Somali region, represented by 12% of the children enrolled, the 5 regions were evenly represented during enrollment of children, with representations ranging between 16% and 19%. Of the total 1905 children enrolled, 39.3% [95% confidence interval (CI): 34.3%, 44.5%] had ≥1 visit from an HEW during the 3 mo before data collection, with the highest contact reported in Amhara region and the lowest in Afar (Table 3). Of the 39.3% children contacted during the past 3 mo, 72% (95% CI: 67.7%, 76.1%) were found to be screened for acute malnutrition. Similar to the contact coverage, majority of the children screened were from Amhara, Sidama, and Southern Nations Nationalities and Peoples' (SNNP) regions; the lowest crude screening coverage was in Afar (Table 3).

**TABLE 3:** Enrollment, contact, and crude screening coverage across the 6 regions

	Total enrolled		Contact coverage^1^			Crude screening coverage^2^		
	*N*	%	%	95% CI		%	95% CI	
				Lower	Upper		Lower	Upper
Total	1905	100.0	39.3	34.3	44.5	72.0	67.7	76.1
Afar	340	17.9	18.9	—	—	30	—	—
Amhara	340	17.9	66.7	—	—	81	—	—
Oromia	320	16.8	22.0	—	—	67	—	—
Sidama	320	16.8	53.9	—	—	80	—	—
SNNP	344	18.1	39.2	—	—	77	—	—
Somali	241	12.7	33.2	—	—	59	—	—

Abbreviations: CI, confidence interval; HEW, Health Extension Workers; SNNP, Southern Nations Nationalities and Peoples'

1 Proportion of children between 6 and 59 mo old who received ≥1 visits from an HEW in the past 3 mo.

2 Proportion of children 6–59 mo who received ≥1 nutritional screening in the past 3 mo from those who had ≥1 visits from an HEW.

**FIGURE 2:**
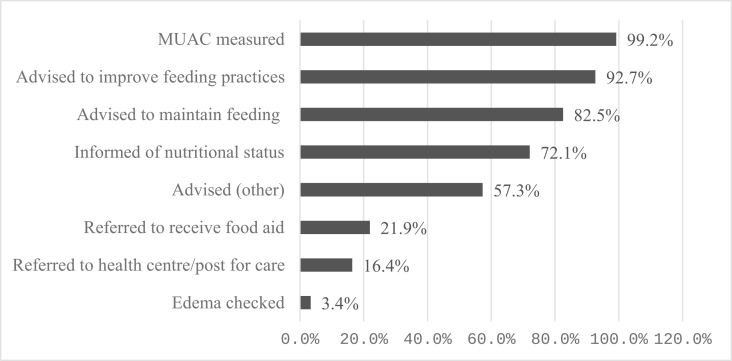
Screening quality index components. MUAC, mid-upper arm circumference.

Figure 2 shows the proportion of children screened and received the recommended need-based clinical actions as reported by the respective caretakers. Most frequently reported clinical actions carried out were MUAC measurement and advice to improve feeding practices when the child was found to have undernutrition, whereas checking for bilateral pitting edema is the least frequently reported clinical action during screening.

To estimate the quality index for screening coverage, the weighted mean for the 8 need-based service variables were calculated, resulting in a quality index of 0.562 (Table 4). The QASC was analyzed based on the formula adopted from Shengelia et al. [18]. With a quality index of 0.562 and a crude screening coverage of 72%, the QASC was found to be 40.4%. The 31.6% difference indicates that nearly half of the screenings performed did not meet the quality standards.

**TABLE 4:** Quality Index (QI) estimation

Need-based variable	*N*	Assigned weight	*n*	% (*n*/*N*)	(Assigned weight)∗*n*/*N*
MUAC measured	958	0.191	950	99.20	18.9%
Edema checked	958	0.191	32	3.38	0.6%
Informed of nutritional status	958	0.191	691	72.08	13.8%
Advised (other)	958	0.191	549	57.33	10.9%
Advised to maintain feeding practices	208	0.041	172	82.54	3.4%
Advised to improve feeding practices	326	0.065	302	92.68	6.0%
Referred to health center/post for care	326	0.065	54	16.44	1.1%
Referred to receive food aid	326	0.065	71	21.92	1.4%
QI (sum total)					56.2%

Abbreviation: MUAC, mid-upper arm circumference.

## Discussion

### Statement of principal findings

The study showed that less than half of the children (39%) in the study area received a visit by an HEW in the preceding 3 mo. Of which, 72% were checked for nutritional status. After adjustment for quality, only 40.4% received the health benefits of the screening program. Significant disparities in coverage were observed across regions, with Afar region showing the lowest coverage.

### Strengths and limitations

The study assesses perceived experience of care takers during routine programmatic nutritional screening. Children from agrarian and pastoralist regions were adequately represented, with a particular focus on rural districts. Furthermore, the large sample size gave the study adequate precision and improved statistical power. However, convenience sampling was used during the selection of regions, which introduced susceptibility for selection bias affecting the generalizability of the findings at the national level. Additionally, the findings are based on self-reported experiences of care takers, which makes the results susceptible to desirability and recall bias.

### Interpretation within the context of the wider literature

Contact coverage was defined as 1 or more visits by an HEW in the 3 mo before data collection. According to the results, only 4 of 10 children had contact with an HEW, regardless of the service provided, during the 3 mo preceding the survey. Different countries follow country-specific guidelines of planning preventive follow-ups for children, depending on their priorities and context. For instance, the American Academy of Pediatrics recommends that children to be visited every 2 mo for their first 6 mo of life, every 3 mo until they are 18 mo old, and every 6 mo after that [19]. However, most LMICs do not have the resources to avail such intensive specialized care for maternal and child health services, and rely on Primary Health Care to provide basic health promotive and disease prevention services, including nutritional screening [20]. Similarly, our results show that children are often not getting the necessary visits from HEWs. The findings are also similar to another study that assessed the geography of public service delivery in rural parts of Amhara, Oromia, SNNP, and Tigray regions, which also showed that only 36% of the households had contacts with HEWs in the 3 mo before the survey [21]. A similar study from Northeastern Ethiopia also reported that HEWs regularly visit only 11.3% of the households, and 50.8% of the households were visited occasionally [22]. All these findings show that the performance of HEWs needs to improve, as contact with HEWs is the primary entry point for health promotion and disease prevention, including nutritional screening. The findings also resulted in a regional variability in contact coverage, evidenced by a relatively higher contact coverage of 66.7% in Amhara region, whereas regions like Afar and Oromia reported fewer visits by HEWs with contact rates of 18.9% and 22%, respectively. According to Miller et al. [23], factors such as lack of awareness of the services offered by HEWs, preference for home-based care, distance, lack of transportation, cost of care seeking, the need to obtain husband’s permission to seek care, health post closures, disrespectful care, and limited skill and confidence of HEWs are some of the hindrances that contribute to lower rates of contacts between HEWs and households. In addition, pastoralist regions such as Afar and Somali exceptionally face multifaceted challenges with access to basic primary health services because of the sparse and scattered settlement patterns of the community, making periodic contact by HEWs a difficult task [24].

Results of coverage levels for quality-adjusted screening showed that for every 10 nutritional screenings, only 4 children benefit. MUAC was the most frequently reported variable (99.2%), whereas checking for bilateral pitting edema was only reported for 3.3% of children screened. This is alarming, as bilateral pitting edema alone signifies the presence of acute malnutrition [2]. Omitting edema measurement has significant implications for enrollment and care of children with acute malnutrition, especially in LMICS, where 62% to 66% of children with edematous malnutrition could have a MUAC or weight-for-height nonindicative of wasting [25]. Checking for bilateral pitting edema is crucial in indicating the presence, severity, and course of action during the management of acute malnutrition. Refresher trainings, mentorship, and community-level interventions should emphasize the importance of checking for bilateral pitting edema and tracking its performance.

The results also show a significant regional variation for crude screening rates. Amhara and Sidama regions have higher rates (81% and 80%, respectively), whereas Afar region has the lowest (30%). A study assessing Growth Monitoring Program in Afar pastoralist districts found only 15.9% service utilization, highlighting challenges because of limited access to primary healthcare [24]. However, a similar pastoralist region, Somali, has a relatively higher screening rate (59%), suggesting the need to consider other health system and sociopolitical factors.

According to the results, only 4 of 10 children who were identified as having acute malnutrition were referred for care and treatment. Lower rate of referral implies that more than half of the children with acute malnutrition and their families are not receiving the expected benefits of the nutritional screening program. This could be due to preference for home-based care, distance, lack of transportation, opportunity cost, the need to obtain husband’s permission to seek care, health post closures, or fear of disrespectful care at health facilities, as reported in other studies [23].

### Implications for policy, practice, and research

Stakeholders must address the alarming gap in assessing children for bilateral pitting edema, a key indicator of acute malnutrition. Further research is needed to explore factors contributing to higher contact rates in Amhara region and lower crude screening coverage in Afar. Moreover, conducting an observational assessment of screening practices can provide valuable insights and validate the findings from this study.

## Conclusion

The study reveals a critical gap in the contact between HEWs and children 6 to 59 mo. Many children miss the health benefits of routine nutritional screening because of suboptimal screening or no screening at all. Only 39.3% of children are visited by HEWs over 3 mo. Moreover, there is significant variation between crude and QASC, indicating a loss of health benefits. Only 4 of 10 children visited by HEWs receive optimal nutritional screening.

## Declaration of generative AI and AI-assisted techologies in the writing process

The author(s) declare that no generative AI or AI-assisted technologies were used in the writing of this manuscript.

## Author contributions

The authors’ responsibilities were as follows – TT, SH, SG, AM, MS, BR, BY: conceived and designed the study; TT, SH, MS: performed the analysis; TT: drafted the manuscript, and all authors: provided critical conceptual revisions, read, and approved the final manuscript.

## Data availability

Data described in the manuscript, code book, and analytic code will be made available upon request, pending application and approval.

## Ethics approval and consent to participate

The ethical considerations involved in this study were carefully observed to protect the rights, welfare, and privacy of the participants and in compliance with national and international ethical standards at all stages of the study. Furthermore, administrative permission was also granted from the Federal Ministry of Health, respective Regional Health Bureaus, and health facilities before data collection. In addition, informed consent was taken from all parents and caretakers before enrolment to the study.

## Funding

This specific research did not receive grant from any nonprofit or for-profit funding entities.

## Conflict of interest

The authors declare that they have no competing interests. No financial or nonfinancial benefits have been received or will be received from any party related directly or indirectly to the subject of this article.
